# *GBA1* Gene Mutations in α-Synucleinopathies—Molecular Mechanisms Underlying Pathology and Their Clinical Significance

**DOI:** 10.3390/ijms24032044

**Published:** 2023-01-20

**Authors:** Zuzanna Granek, Julia Barczuk, Natalia Siwecka, Wioletta Rozpędek-Kamińska, Ewa Kucharska, Ireneusz Majsterek

**Affiliations:** 1 Department of Clinical Chemistry and Biochemistry, Medical University of Lodz, Mazowiecka 5, 92-215 Lodz, Poland; 2 Department Geriatrics and Social Work, Jesuit University Ignatianum in Cracow, Kopernika 26, 31-501 Cracow, Poland

**Keywords:** *GBA1* mutations, glycosylceramidase, glucocerebrosidase, Gaucher’s disease, α-Synuclein, α-synucleinopathies, Parkinson’s disease, dementia with Lewy bodies, multiple system atrophy

## Abstract

α-Synucleinopathies comprise a group of neurodegenerative diseases characterized by altered accumulation of a protein called α-synuclein inside neurons and glial cells. This aggregation leads to the formation of intraneuronal inclusions, Lewy bodies, that constitute the hallmark of α-synuclein pathology. The most prevalent α-synucleinopathies are Parkinson’s disease (PD), dementia with Lewy bodies (DLB), and multiple system atrophy (MSA). To date, only symptomatic treatment is available for these disorders, hence new approaches to their therapy are needed. It has been observed that *GBA1* mutations are one of the most impactful risk factors for developing α-synucleinopathies such as PD and DLB. Mutations in the *GBA1* gene, which encodes a lysosomal hydrolase β-glucocerebrosidase (GCase), cause a reduction in GCase activity and impaired α-synuclein metabolism. The most abundant *GBA1* gene mutations are N370S or N409S, L444P/L483P and E326K/E365K. The mechanisms by which GCase impacts α-synuclein aggregation are poorly understood and need to be further investigated. Here, we discuss some of the potential interactions between α-synuclein and GCase and show how *GBA1* mutations may impact the course of the most prevalent α-synucleinopathies.

## 1. Introduction

α-Synucleinopathies comprise a group of neurodegenerative diseases characterized by altered accumulation of a protein called α-synuclein inside the neurons and glial cells [1]. α-Synuclein is an α-helical protein widely expressed in the Central Nervous System (CNS). The exact function of α-synuclein is still poorly understood, however, mostly due to its presynaptic localization, it is suggested that it might be associated with modulating synaptic function, such as synaptic plasticity and activity, as well as vesicle trafficking [2].

The most prevalent α-synucleinopathies constitute Parkinson’s disease (PD), dementia with Lewy bodies (DLB) and multiple system atrophy (MSA) [1]. Although these diseases vary between each other in severity, symptoms and prevalence, they all affect the autonomic nervous system. Some of the most common symptoms regarding α-synucleinopathies comprise urinary and sexual dysfunction, constipation and cardiovascular autonomic symptoms. Interestingly, symptoms associated with autonomic dysfunction often develop before the onset of motor symptoms [1].

DLB is the second most common cause of dementia worldwide, accounting for 10–15% of all its cases. The connection between *DLB* and *GBA1* gene mutations has been confirmed. Similar to *DLB*, *GBA1* gene variants are regarded as the most significant risk factor for PD development and around 5–30% of all PD patients carry GBA1 mutations. The association between GBA1 mutations and MSA, however, is poorly understood and there are conflicting findings regarding their potential connection.

In this review, we outline the most abundant GBA1 mutations, highlight the link between GBA1 gene variants and the courses of most prevalent α-synucleinopathies, as well as discuss the mechanisms by which dysfunction of glucocerebrosidase (*GBA1* gene product) might impact α-synuclein accumulation and α-synucleinopathies progression.

## 2. *GBA1* Gene Mutations and the Associated Pathologies

### 2.1. The Structure and Function of the GBA Gene and Its Protein Product, Glucocerebrosidase

#### 2.1.1. The GBA Gene

The *GBA1* gene (glucosylceramidase beta) is a protein coding gene located on chromosome 1 (1q21). It is currently known that the size of the *GBA1* gene is approximately 7.6 kb and it is composed of 13 exons. The *GBA* pseudogene’s (GBAP) size is approximately 5.7 kb and, according to new findings, it is located 6.9 kb downstream of GBA1 [3]. The sequences of GBA1 and GBAP1 are 96% identical and show structural similarity in exon–intron organization. Introic Alu sequence of transposon and a 55bp deletion in exon 9 surrounded by an inverted short repeat differentiate between pseudogene and *GBA1* gene [4,5]. According to the newest report, GBAP1 may be involved in the regulation of GBA1 expression [3]. Recently, it was reported that the *GBA* gene has distal (P1) and proximal (P2) promoters which have multiple splicing variants and transcription start sites (TSS) [3]. On the other hand, *GBA2* and *GBA3* genes are located on chromosome 9 and 4, and contain 18 and 6, exons, respectively [6,7].

To date, it is known that GBA1, GBA2 and GBA3 encode three distinct glucosylceramidases. GBA1 product catalyzes the hydrolysis of glucosylceramides into free ceramides and glucose in the lysosome, and it is associated with Gaucher’s disease type I and II (GD), Parkinson’s disease (PD) and dementia with Lewy bodies (DLB) [8,9]. GBA2 encodes a microsomal β-glucosidase that catalyzes the hydrolysis of bile acid 3-O-glucosides and it is associated with spastic paraplegia 46 and autosomal recessive cerebellar ataxia with late-onset spasticity [10,11]. GBA3 is a cytosolic polymorphic pseudogene, with the most common allele encoding the full-length protein. GBA3 mutations are associated with GD; however, it was reported that they do not seem to modify type 1 GD manifestation [12]. This review concentrates on *GBA1* as the most important gene in the pathogenesis of α-synucleinopathies.

#### 2.1.2. GBA1 Product—Glucocerebrosidase

The *GBA1* gene encodes a ubiquitously expressed hydrolase, β-glucocerebrosidase (GCase). GCase catalyzes the cleavage of glycosphingolipids–glucosylceramide (GlcCer) and glucosylsphingosine (GlcSph) to glucose and ceramide, and to glucose and sphingosine, respectively [13]. The optimal pH for the enzyme activity is 4–5 [14]. In contrast to other lysosomal proteins, GCase is targeted to the lysosomes by the lysosomal integral membrane protein-2 (LIMP-2), the mannose-6-phosphate-independent trafficking receptor for β-glucocerebrosidase [15]. GCase attaches to a coiled-coil domain in the LIMP-2 lumenal region and, together with associated proteins, it passes through the endosomes and Golgi apparatus into the acidic environment of lysosome where they dissociate. Phosphatidylinositol 4-kinase type IIIb (PI4KIIIb) is needed for the exit of the sequentially traversing GCase-LIMP-2 complex from the Golgi body, and the PI4K type IIa (PI4KIIa) is required for the proper sorting of the complex from endosomes [16]. For the proper rates of glucosylceramide hydrolysis, the interaction with sphingolipid activator proteins (SAP-2), Saponin C and negatively charged lipids is necessary [17,18].

**Figure 1:**
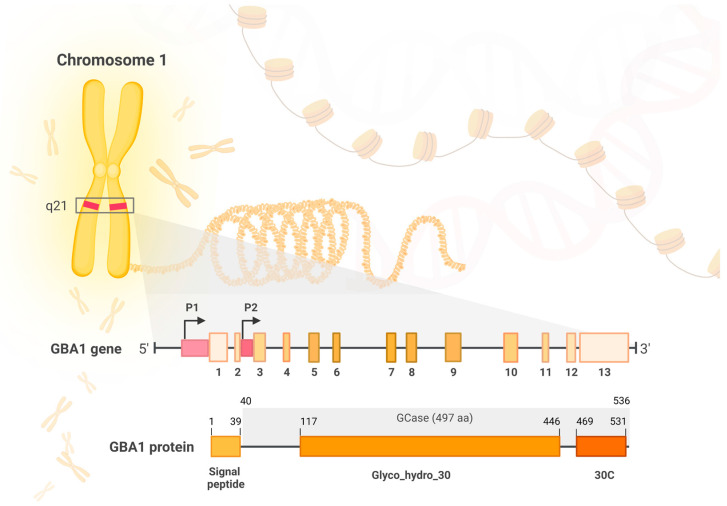
The structure of the *GBA1* gene and the encoded GBA1 protein (β-glucocerebrosidase/GCase). *GBA1* gene, located at q21 region of chromosome 1, comprises 13 exons and 2 promoters (P1 and P2), and is characterized by multiple splicing variants and transcription start sites. GBA1 protein is composed of three main regions: 39-residue signal peptide, the conserved catalytic domain Glyco_hydro_30 (329 aa) and Glyco_hydro_30C domain (30C in the picture; 62 aa). The mature GBA1 protein—GCase—is composed of 497 amino acids (residues 40–536) and it includes Glyco_hydro_30 and 30C regions.

The functional GCase protein consists of 497 amino acids and has a size of approximately 59 and 69 kD, which depends on its post-translational modifications [19]. The enzyme comprises three intermittent regions—domain I—an antiparallel β-sheet, domain II—a (β/α)8 triosephosphate isomerase (TIM) barrel which shelters the active site, and domain III—an eight-stranded β-barrel, resembling an immunoglobulin fold [20] (Figure 1).

### 2.2. GBA1 Gene Mutations (Table 1)

Mutations in the *GBA1* gene cause a reduction in GCase activity and, due to progressive lysosomal dysfunction, this results in impaired α-synuclein metabolism. Accumulation of pathological forms of α-synuclein leads to the formation of intraneuronal inclusions named Lewy bodies, which are a key hallmark of α-synucleinopathies.

It is assumed that high homology and close proximity between *GBA1* and *GBAP1* genes increase the risk for allelic recombination (both reciprocal and non-reciprocal) and promote the formation of complex alleles. This may result in a generation of gene duplications, fusions and conversion [5]. Many different mutations in GBA1 such as point mutations, deletions, insertions, frameshift missense, concomitant multiple mutations, splice junctions and null alleles have been reported [21]. The characteristic variants can be distinctly represented in particular phenotypes as well as in specific ethnic groups. Due to the fact that pathogenic mutations are located throughout the entire protein, the distance of the mutations to the active site cannot be a reliable predictor of severity of the disease [22]. Main *GBA1* gene mutations include c.1226A>G (N370S or N409S in new nomenclature), c.1448T>C (L444P/L483P) and c.1093G>A (E326K/E365K). N409S mutation is located on the interface of domains II and III [23]. Firstly, it induces loss of GCase activity, then activation of the unfolded protein response (UPR) signaling pathway and α-synuclein pathology. This mutation may affect the stability of the helical turn conformation of loop 1. It results in lower disease penetrance and a milder clinical phenotype [24]. The L483P mutation is situated on domain II and causes altered lipid profile as an indicator of increased risk for α-synucleinopathy. It promotes higher disease penetrance and a worse clinical phenotype [25]. Both N409S and L483P mutant variants present reduced capacity of GCase to interact with its activator, Saposin C, and with anionic phospholipids [26].

The E365K variant affects the surface of domain III, reduces GCase activity to a lesser extent than GD-causing mutations and defines a worse clinical phenotype [27].

#### 2.2.1. GBA1 mutations in Gaucher’s Disease

The biallelic mutation of GBA1, a recessively inherited deficiency of the GCase, directly causes GD [22,26]. This is the most common lysosomal storage disorder and is associated with more than 495 mutations of the *GBA1* gene [21]. The disease results from the accumulation of glucocerebrosides in the lysosomes of macrophages named ‘Gaucher cells’, which can be characterized by deregulated expression of cell surface markers, iron sequestration, abnormal secretion of inflammatory cytokines and ability to infiltrate tissues; this results in hematological manifestations, splenomegaly and bone diseases [28]. There are five known types of GD: type 1, type 2, type 3, perinatal lethal and cardiovascular, that are distinguished on the basis of the neuronal involvement and the resulting severity of symptoms. [29]. GD type I is non-neuronopathic and accounts for 95% of cases, that are characterized by pancytopenia, splenomegaly and osteoporosis. Type II is acute neuronopathic and type III is chronic neuronopathic—these types demonstrate progressive neurological deterioration [30]. Manifestation of GD occurs when a patient is a carrier of a pathogenic mutation on both alleles of the *GBA1* gene, either as a homozygote or heterozygote. GBA1 mutations are classified depending on which type of GD they may cause. Severe mutation must be inherited from both parents and it results in severe phenotype of GD types II or III, characterized mainly by a more aggressive disease course and in vitro residual GCase enzymatic activity of 13–24% [31]. When mutation is inherited in a compound heterozygous or homozygous manner, then it is called mild mutation with GCase activity of 32–38% in vitro and it causes the mild type of GD (type I) [32,33,34]. On the other hand, the most common mutations of the *GBA1* gene are N409S and L483P. Homozygosity for N409S mutation is associated exclusively with GD type I patients in the USA, Europe and Israel, while the L483P mutant type (MT) is seen in GD type III patients worldwide [35,36]. The newest treatment of GD includes mainly enzyme replacement therapy (ERT), substrate replacement therapy (SRT), chaperone molecules, eliglustat, matched sibling hematopoietic stem cell transplantation and gene therapy [37,38,39].

#### 2.2.2. The Association between Gaucher’s Disease and Parkinson’s Disease

The hint about the association between GD and PD was based on the clinical observation that 25% of GD patients report to have a first- or second-degree relative suffering from parkinsonism [33,40]. Subsequently, a huge, multi-center study of European, American, Asian and Israeli patients analyzed genotypes and phenotypic data from a total of 5691 PD subjects of diverse ethnic origin and 4898 controls, demonstrating a strong association between GBA1 MT and PD [41]. Penetrance of GBA1 mutations is variable (10–30%) and age dependent. A cumulative risk of developing PD in 60- and 80-years-of-age subjects is, respectively, 5% and 10–30% in heterozygous GBA1 mutation carriers compared to 1–3% in non-carriers [41,42,43,44,45]. Homozygous GBA1 variant-linked patients who are affected by GD have a 10-times greater risk of developing PD and an earlier age of symptom onset [45,46]. It has been shown in a wide array of clinical studies that around 9.1% of GBA1 mutation carriers will develop PD [35].

#### 2.2.3. GBA1 Mutations in Parkinson’s Disease

According to a huge multicenter analysis by Sidransky et al., approximately 5–30% of patients who suffer from PD have GBA1 mutations [41]. In the mentioned report, it was for the first time definitively established that GBA1 mutations are five times more frequent in PD patients than controls. The highest reported prevalence of the GBA1 mutation was ~20% for Ashkenazi Jewish patients, whilst in non-Ashkenazi patients, it was estimated for ~7%. The major differences in the results obtained were due to ethnicity of the studied populations, the number of screened mutations and the extent of exome sequencing. It should be noted, however, that overall incidence of GBA1 mutations was increased in all PD groups studied, regardless of the ethnicity of the participants or mutation type. Moreover, recent genome wide association studies have confirmed that approximately 8–12% of patients who suffer from PD around the world have GBA1 mutations [47].

The disease is called GBA1-associated PD (GBA1-PD). Due to their more frequent occurrence than mutations of other genes associated with familial PD (e.g., LRRK2, SNCA and PARK2), variants of *GBA1* gene are regarded as the most significant genetic risk factor for PD. In the standard classification based on the phenotypic effects in GD, the GBA1 variants are divided into severe, mild, complex and risk. Complex variants result from fusions, conversions and insertions of parts of highly homologous pseudogene *GBAP1* into GBA1. Risk variants are those that increase the risk of PD, but do not induce GD pathology [44].

Around 130 GBA1 mutations were reported in PD patients [36,48]. Similar to GD, L483P and N409S are the two most crucial mutations among others [21]. Severe mutations such as L483P, IVS2 + 1, L29Afs*18, V394L, RecNciI and D409H are correlated with a higher risk of developing PD compared to mild mutations such as N409S and L29Afs*18. [49]. Moreover, severe mutations have a higher incidence, they increase cognitive decline and risk of dementia and are linked to earlier age of onset [42,50]. In addition, the T369M allele was associated with an increased risk for PD [51]. Compared with PD patients with mild GBA1 mutations or idiopathic PD (iPD), subjects with extreme GBA1 mutations show distinctly worse motor and non-motor symptoms, such as insomnia and rapid-eye-movement (REM) sleep disturbances. Results of the recent meta-analysis indicate the higher female prevalence with ethnic specificity and younger age of onset in GBA1-PD patients [52]. Several variants have an unclear role in GD pathogenesis, but constitute risk factors for PD, with the most notable example of E365K mutation [53]. This indicates the potential existence of various mechanisms by which mutations make their carriers vulnerable to PD. Interestingly, GBA1 mutations reveal specific ethnic heterogeneity [36]. Heterozygous GBA1 mutations, as a pivotal genetic risk factor for PD, were confirmed across different ethno-racial populations with Asian (Chinese, Japanese, Taiwanese), Caucasian, African and Hispanic ancestry [54,55,56,57,58]. N409S conveys a panethnic PD risk, R496H and 84insGG is a common PD risk factor in the Ashkenazi Jew (AJ) population, L483P, E365K, T369M, IVS2+1G>A, RecNciI (4856_4905), R159W, H294Q—in non-AJ populations, whereas E365K, H294Q, D409H—in European/West Asians, R120W—in East Asians [41].

#### 2.2.4. GBA1 Mutations in Dementia with Lewy Bodies

The association between GBA1, APOE (apolipoprotein E), SNCA (α-synuclein) gene mutations and DLB was indisputably confirmed. Variants of the mentioned genes modulate risk for the development of the specific DLB phenotypes [59,60,61,62]. Heritability of DLB is estimated at 36% [61]. In many studies, it has been proven that GBA1 MT individuals exhibit an increased risk of developing DLB, notably higher than that for PD [60,61,63]. GBA1 variants increased the risk of DLB development, especially N409S, E365K and L483P which are strongly associated with DLB, but T369M, on the other hand, did not [64]. The E365K mutation is also frequently found in patients with PD dementia (PDD) [65]. GBA1 expression profiles were shown to be reduced in the temporal cortex in DLB and in the caudate nucleus in PDD, as well as in the peripheral blood in both PD and DLB patients [66]. The genetic correlation between DLB and AD was 0.578, and between DLB and PD was 0.362 [61].

**Table 1:** The most common *GBA1* mutations, their prevalence in various populations and association with the specific pathologies.

Common Variant Name (New Nomenclature)	DNA Nucleotide Change	Ethnicity	Disease	Reference
N370S (N409S)	c.1226A>G	AJ *, Non-AJ *, Russian, North African	GD type 1; PD;	[21,41,64,67,68,69,70]
L444P (L483P)	c.1448T>C	Chinese, Japanese, Caucasian, Canadian, Italian, Brazilian, Greek, Non-AJ, AJ, North African	GD type 2 or 3 or neuropathic; PD; DLB	[41,64,67,70,71,72,73,74]
E326K (E365K)	c.1093G>A	Non-AJ, AJ	PD; DLB; RBD	[41,53,64,67]
T369M	p.T369M substitution	Non-AJ, AJ	PD; RBD	[41,51,67]
Rec*Nci*I (4856_4905)	A456P, V460V recombinant	Non-AJ, AJ, Japanese, North African	PD	[41,67,70,75,76]
84GG (L29Afs*18)	c.84dupG	AJ	PD	[41]

* Ashkenzai Jews—AJ; non-Ashkenzai Jews—non-AJ; Gaucher’s disease—GD; Parkinson’s disease—PD; dementia with Lewy bodies—DLB; REM sleep behavior disorder—RBD.

### 2.3. The Effects of GBA1 Mutations on GCase Activity

**Figure 2:**
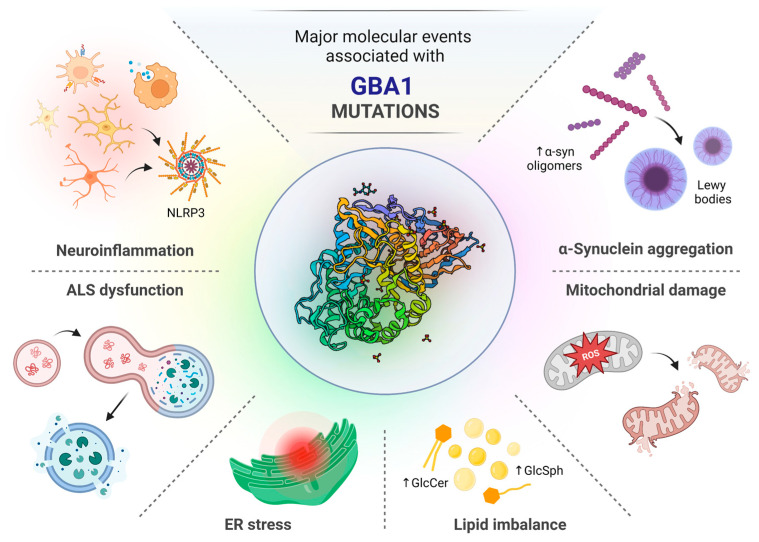
The schematic representation of major molecular consequences induced by GBA1 mutations. The structure of the GBA1 protein is presented in the middle (PDB ID: 1OGS). Mutations in GCase usually result in significant decrease in enzyme activity, that in turn greatly impacts the functionality of autophagy–lysosomal system (ALS); this affects lipid homeostasis due to accumulation of glucosylceramide (GlcCer) and glucosylsphingosine (GlcSph) as well as facilitates aggregation of α-synuclein (α-syn). Excessive accumulation of lipids and abnormal proteins within the cell induces endoplasmic reticulum (ER) stress conditions and cause damage to the mitochondria. All mentioned events lead to induction of neuroinflammatory response with subsequent activation of NLRP3 inflammasome.

Various GBA1 mutations influence the enzymatic activity of GCase in distinct ways. Many of them decrease or even abolish the residual activity of the enzyme [77]. It has been shown that GBA1 homozygotes/compound heterozygotes have lower GCase enzymatic activity as compared to GBA1 heterozygotes and GBA1 and LRRK2 non-carriers. Such reduction in GCase enzymatic activity, especially observed in substantia nigra pars compacta (SNpc) [78], is strongly associated with GBA1 mutations and modestly associated with idiopathic PD. Thus, it can be assumed that a decrease in GCase function in both GBA1 mutation carriers and non-carriers contributes to PD pathogenesis in a wider PD population [47,79]. Moreover, a recent study indicated that LRRK2 kinase activity affects the catalytic activity of GCase in a cell-type-specific manner, giving valuable implications for therapeutic application of LRRK2 inhibitors in GBA1-linked and iPD cases [80]. It has been established that GBA1 mutation severity correlates inversely with GCase activity [13]. The newest data indicate that the severity of α-synucleinopathies is not only related to the level of GCase activity, but also to the fact that mutated GCase is retained in the ER and not trafficked to lysosome. Restricted, perturbed transport of GCase to lysosome affects the autophagic–lysosomal pathway (ALP) and is linked to aggregation of α-synuclein [15]. The existence of invalid GCase protein can induce the UPR signaling pathway and endoplasmic reticulum (ER) stress conditions [14]. Dysfunction of mitochondria, lipid homeostasis and specific inflammation profile have also been reported in both GBA1-PD and DLB, as they may also lead to α-synuclein-related pathology [18,19]. Post-translational modifications of α-synuclein including phosphorylation, ubiquitination, truncation, nitration and O-GlcNAcylation impair the lysosomal-mediated degradation of the protein, thereby contributing to its accumulation [81] (Figure 2).

### 2.4. A Correlation between GCase Activity and α-Synuclein Accumulation

A link between α-synuclein and GBA1 was suggested when intraneuronal α-synuclein inclusions were observed in GD type 1 patients with parkinsonism [20,82]. An inverse correlation between GCase activity and α-synuclein accumulation has been reported in GCase-deficient cells, fly and mouse models, as well as in GBA1-PD and sporadic PD brains [77,82,83]. Overexpression of α-synuclein can induce, among others, vascular pathology, blood brain barrier leakage and pericyte activation [84]. Not only was it observed that overexpression of α-synuclein results in decreased GCase activity, but also that enhancing GCase activity can rescue α-synuclein pathology. The bidirectional loop between α-synuclein and GCase rises from the fact that deficient GCase leads to the accumulation of substrates bound by α-synuclein and that α-synuclein itself can lower the enzymatic activity of GCase [17,85]. It was proven in a transgenic mouse model that pathological α-synuclein fibrillar forms decrease GCase activity, which induces neuronal susceptibility to neurodegeneration [18]. Recently, co-cultures of astrocytes and dopaminergic neurons from GD type 1 and 2 patients revealed reduced GCase activity, impaired Cathepsin D activity and significant α-synuclein accumulation when treated with α-synuclein fibrils and monomers, that contributed to neuroinflammation [16,86]. Recently, human midbrain-like organoids (hMLOs) deficient in GCase and coupled with wild-type α-syn overexpression, accumulated Lewy body-like inclusions which were absent in organoids with GCase deficiency or SNCA triplication alone. This demonstrated that impaired GCase function promotes α-synuclein pathology. It has been established that GCase deficiency and wild-type α-syn overproduction are the two major risk factors for PD [26]. Furthermore, it has been proved in animal models and clinical trials that small molecule chaperones, such as ambroxol, isofagomine, NCGC607 and S-181, can restore the levels of GCase and α-synuclein in PD neurons [77].

## 3. α-Synucleinopathies Associated with GBA1 Mutations—Clinical Characteristics

### 3.1. Parkinson’s Disease

Clinically, GBA1-PD mirrors iPD. However, carriers of the GBA1 mutation exhibit earlier disease onset, reduced survival, greater family history, more severe and frequent motor symptoms (dyskinesias, dysphagia, dysarthria, freezing of gait) and non-motor symptoms (dementia, visual hallucinations, depression, anxiety, RBD, olfactory dysfunction) [87]. They are also characterized by more severe cognitive impairment and neuroimaging features, such as impaired cortical activity and nigrostriatal function [36,88]. The genotype–phenotype correlations within GBA1-PD patients can be observed in diverse clinical features which need more variant-based investigation to elucidate the influence of specific GBA1 variants on the PD course. The more rapid decline in motor and non-motor features in GBA1-PD patients should be considered in the context of personalized treatment strategies, e.g., individualized pharmacological treatment, physiotherapy or cognitive engagement strategies at the early course of the disease [89].

For PD patients harboring the GBA1 mutation, the mean age of onset is 56.8 years (median: 58, range: 30–79), which is 3 to 6 years earlier compared to iPD [67]. Disease manifests about 6–11 years earlier in homozygous mutation carriers and about 3–6 years earlier in subjects with heterozygous mutations, regardless of the severity of the mutation [42,88,89,90]. A cumulative risk of developing PD in 60- and 80-year-old GBA1 heterozygous mutation carriers is 5% and 10–30%, respectively, when compared to non-carriers [42,44,91]. Multivariable analysis adjusted by sex, age of onset and disease duration in GBA1 mutation carriers reported a reduced survival compared to non-carriers (HR = 1.85; *p* = 0.002) [92].

#### 3.1.1. Clinical Features

Among GBA1-PD patients, the rigid akinetic phenotype is more common and accompanied by tremor and bradykinesia. Usually, these patients respond very well to levodopa, although the deterioration during disease course and progression of the motor symptoms is slightly faster compared to iPD, but without higher rates of motor fluctuations [93]. Motor complications, such as dyskinesia, dysphagia, dysarthria, freezing of gait and axial symptoms such as postural instability occur earlier and are more frequent in GBA1 MT carriers, especially in those harboring severe mutations [36,50,94,95,96]. Interestingly, non-pathogenic GBA1 variants and polymorphisms are associated with a greater risk of motor deterioration and may affect motor symptomatology [93,94]. In recent studies, the most beneficial treatment on the motor symptoms was evaluated with deep brain stimulation (DBS) in a cohort of GBA1-PD patients compared to iPD, albeit cognitive impairment and non-motor symptoms did not improve [89,97].

PD patients with GBA1 mutations exhibit up to three times higher risk of developing cognitive decline compared to iPD. This affects visual short-term memory, executive and visuospatial functions and dementia [45,91,98]. On the contrary to other PD symptoms, the degree of cognitive impairment is correlated with the severity of GBA1 mutation and GCase activity [27,50,99,100]. Deficient α-synuclein clearance mechanisms in GBA1 mutants leading to intensified α-synuclein accumulation in cortical areas is considered to exacerbate cognitive impairment and, in the long run, induce dementia in GBA1-PD patients [94]. A subtle alteration in cognitive functioning in GBA1 mutation-positive individuals without PD was also confirmed [47,101]. It has been found that GBA1-PD had a greater prevalence of depression (33.3%) versus iPD (13.2%) (*p* < 0.05) [102]. The newest study has reported that GBA1-PD patients with depression showed statistically valuable decreased fractional anisotropy or increased mean diffusivity in the specific brain regions compared with matched iPD patients with depression. It is assumed that depression in GBA1-PD is associated with microstructural damages in the limbic system [103].

Olfactory function worsens and tends to deteriorate over time in GBA1-PD resulting in hyposmia, especially in patients carrying pathogenic severe variants of GBA1 [89,103,104]. Autonomic symptoms such as constipation, urogenital dysfunction, gastrointestinal symptoms, orthostatic hypotension and sexual dysfunctions have frequently been reported in GBA1-PD patients [42,94,105]. RBD are also more frequent among subjects with GBA1 homozygous mutations and in patients carrying severe vs. milder mutations [8,94,106]. Increased frequency and risk of psychiatric symptoms, such as hallucinations, delusions, and impulsive–compulsive behavior, has also been reported in GBA1-PD patients vs. non-carriers [50,105,107]. The prevalence of hallucinations in GBA1 MT carriers remains four times higher than in GBA1 non-carriers [95].

#### 3.1.2. Neuroimaging Features

GBA1–PD patients exhibit deregulation of the presynaptic dopamine terminal function observed as significant dopamine transporter (DAT) deficit, mainly in the striatum contralateral to the more affected side [42]. Investigation of metabolic networks in GBA1-PD patients using [18F]-FDG PET (fluorodeoxyglucose-positron emission tomography) has shown increased PD-related pattern (PDRP), PD-related cognitive pattern (PDCP) levels and significant [18F]-FDG PET hypoactivity in the parietal lobe, reflecting higher cognitive burden compared with non-carriers [108,109]. However, it has been reported that N409S, E365K and T369M variant carriers have reduced [18F]-FDopa uptake in the bilateral caudate nuclei, ipsilateral anteromedial putamen and contralateral nucleus accumbens to the more affected side [109,110,111]. It is yet to be established whether the slower decline rate in DAT signal in GBA1-PD subjects is caused by the compensatory upregulation of tracer uptake in the early stage of the disease or disruption of dopamine release preceding dopaminergic terminal loss [110,111]. In addition, enlarged hyperechogenic area within substantia nigra and interrupted brainstem raphe (a marker of serotonergic system impairment) were detected in transcranial sonography in GBA1 mutation carriers compared with healthy controls [105,112]. Furthermore, local brain atrophy was reported in GBA1-PD patients resulting directly in varying intensity of cognitive decline [113,114]. Advanced neuroimaging techniques such as MRI, SPECT (single-photon emission computerized tomography), 18F-FDG PET and brain perfusion studies have shown more aggressive disease course and greater cortical involvement in GBA1-PD compared to iPD patients. The hallmark conclusion is that cognitive impairment which stands as a ‘clinical signature’ of GBA1-PD, seems to have its neuroimaging correlation in greater burden of cortical region in these patients compared to iPD [115].

### 3.2. Dementia with Lewy Bodies

DLB is an α-synucleinopathy that is the second most common cause of degenerative dementia, following Alzheimer’s disease (AD). It is regarded as an underlying etiology of 10–15% of all cases of dementia. It shares genetic, pathological and clinical features with PD and AD; however, DLB is thought to have a shorter disease duration and decreased survival rate compared to AD [116]. Recent meta-analyses reported that sex is irrelevant to DLB development [64]. The microscopic hallmark of DLB is intracellular inclusions composed by α-synuclein, ubiquitin, neurofilaments, α-crystallin B and valosin-containing protein, which are thought to induce the progressive loss of structure and functions of neurons. Microtubule regression and mitochondrial loss leads to decreased cellular energy and axonal transport, ultimately ending up in the death of neurons [117]. The pathology of TAR DNA-binding protein 43 (TDP43), phosphorylation of tau, microglia and T-lymphocyte recruitment, as an inflammatory response, also plays a key role in the pathology of DLB [19,118,119,120]. According to the newest report, GBA1 mutation carriers have more severe DLB symptoms and purer neuropathological lesions [121].

#### 3.2.1. Clinical Features

Patients harboring GBA1 variants have an approximately five years earlier age of DLB onset, increased hallucinations, worse REM sleep behavior disorder (RBD) symptoms, more severe motor and cognitive impairment and rapid symptom progression compared to non-carriers [60,122,123]. Among GBA1 mutant carriers, the risk of developing DLB is about three times greater than for PD [124].

The core clinical features of DLB are fluctuation in cognition and attention, recurrent visual hallucinations and RBD occurring early in the disease course, as well as spontaneous parkinsonian motor signs, which often occur later. If parkinsonism precedes the onset of dementia by a year or more, the diagnosis of Parkinson’s disease dementia (PDD) is made, whereas if parkinsonism occurs at the same time or within 1 year of dementia, DLB is diagnosed. Lewy body dementia encompasses both DLB and PDD [63]. Supportive clinical features of DLB include sensitivity to antipsychotic agents, postural instability, repeated falls, syncope or other transient episodes of unresponsiveness, severe autonomic dysfunction, hypersomnia, hyposmia, hallucinations, apathy, anosognosia, anxiety and depression [121,125,126].

In contrast to neuronal loss primarily located in the substantia nigra, resulting in a predominant motor clinical manifestation in PD course, in DLB, Lewy bodies are found mainly in the neocortex and limbic system and then they propagate downwards to the brainstem [127].. Lewy bodies’ deposition in the midbrain, which results in orthostatic hypotension, may induce autonomic failure that, together with motor symptoms, contributes to a high risk of falls. The effect of α-synuclein on the enteric nerve cells or autonomic dysfunction as seen in PD may directly lead to constipation. Interestingly, it has been proven that misfolded α-synuclein can transfer between cells and, once trafficked into a new cell, can recruit endogenous α-synuclein, leading to the formation of larger aggregates, similarly to prion disease [128,129].

#### 3.2.2. Neuroimaging features

The best-established neuroimaging method for the diagnosis of α-synucleinopathies is dopaminergic 123I-FP-CIT SPECT scanning, which can demonstrate a reduction in the levels of dopamine transporter in striatal neuronal pathways, in the bilateral caudate nucleus and in the putamen, both in individuals with PD and DLB [130,131]. Biomarkers for DLB detection include abnormally low uptake on 123I-MIBG in myocardial scintigraphy and REM sleep without atonia in polysomnography, relative preservation of medial temporal lobe structures on MRI or CT scan, generalized low uptake on DAT-SPECT or PET perfusion–metabolism scan and prominent posterior slow-wave activity on EEG with periodic fluctuations in the pre-α and/or theta range [131,132,133,134].

The early recognition and diagnosis of DLB has critical treatment implications. The treatment of DLB includes cholinesterase inhibitors for cognitive and behavioral impairment symptoms of DLB: rivastigmine, galantamine and donepezil, memantine, atypical antipsychotic drugs for agitation, levodopa/carbidopa for parkinsonism, pimavanserin for psychosis, melatonin or clonazepam for RBD and personalized psycho-cognitive treatment [135,136,137].

### 3.3. Multiple System Atrophy

Although all PD, DLB and MSA often share common genetic risk factors, the connection between GD and MSA is unclear and studies did not find a correlation between GD variants and MSA. In contrast to that, previous research on large case–control groups has shown that there might, in fact, be a correlation between some GD variants and increased MSA risk [138]. However, there are still some conflicting findings on this subject and the actual link between the *GBA1* mutation and MSA is yet to be determined [139].

## 4. Mechanisms Underlying the Crosstalk between GCase and α-Synuclein

The mechanisms by which GCase impacts α-synuclein aggregation are multifaceted and still poorly understood. These two molecules are suspected to mostly interact in acidic pH and to co-localize in the lysosome. Therefore, this organelle has been suggested to be the primary site of α-synuclein and GCase interaction [140]. One important aspect is that in the presence of mutant GCase in the lysosome, the levels of its product, ceramide, become reduced. Mechanistically, ceramide induces maturation of cathepsins (a group of lysosomal proteases that degrade, inter alia, α-synuclein), and lower levels of ceremide considerably impair lysosomal function. This results in decreased degradation and higher levels of α-synuclein, and therefore higher risk for developing PD. Increased α-synuclein levels further inhibit GCase activity, which ultimately creates a vicious cycle [141]. Interestingly, a study evaluating the correlation between α-synuclein pathology and GCase activity found that the inhibition of GCase does not trigger α-synuclein de novo aggregation or upregulate total α-synuclein levels in transgenic mice [142]. However, GCase inhibition does result in increased α-synuclein levels upon the pathology initiation with misfolded α-synuclein seeds. Hence, it is claimed that GCase impact on α-synuclein pathology does not depend on the neuron type but rather on the extent of the existing pathology [18].

**Figure 3:**
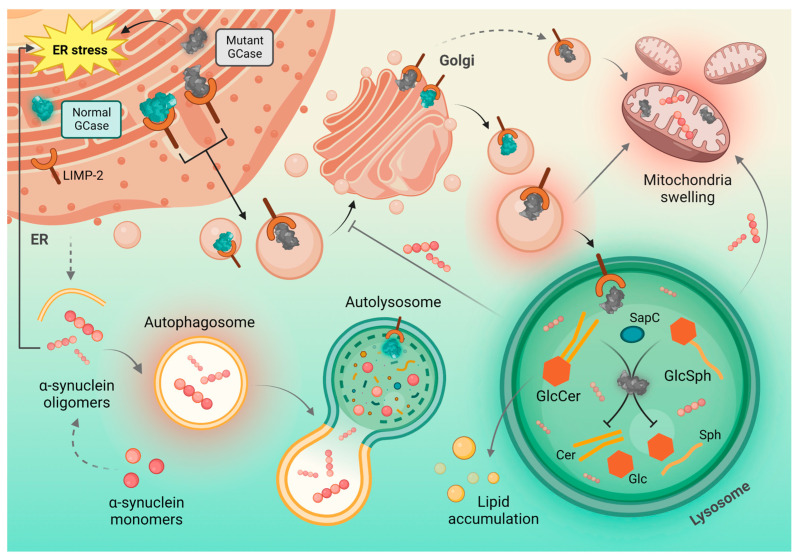
Molecular mechanisms underlying GCase mutations and the associated cellular events. Under normal conditions, the functional GCase is synthesized in the ER, bound to lysosomal integral membrane protein type-2 (LIMP-2) and transferred to the Golgi apparatus for glycosylation. Then, it is targeted to the lysosomes, where it catalyzes degradation of glucosylceramide (GlcCer) and glucosylsphingosine (GlcSph) to glucose and ceramide or glucose and sphingosine, respectively; Saposin C (SapC) acts as a cofactor in this process. However, in the case of mutant GCase, the activity of enzyme is markedly decreased, which leads to accumulation of GlcCer and GlcSph, and in consequence—lipid imbalance. Impaired function of GCase also affects the degradation of other lysosomal substrates, such as α-synuclein oligomers. Accumulation of mutant GCase and α-synuclein within the cell contribute to endoplasmic reticulum (ER) stress, as well as mitochondria swelling and dysfunction. Moreover, α-synuclein aggregates inhibit the GCase transport from ER to Golgi, which further decreases GCase activity and generates a bi-directional positive feedback loop.

α-Synuclein aggregates contiguous propagation can also result from lysosomal dysfunction, which is a potential secondary effect of *GBA1* function loss [143]. Nevertheless, other molecular mechanisms, described later in this article, such as disturbed protein or lipid metabolism, ER stress or mitochondrial dysfunction also substantially contribute to the pathology progression in patients with GBA mutations (Figure 3).

### 4.1. Lipid Metabolism

Alterations in lipid levels associated with decreased GCase activity have been suggested to impact the ability of α-synuclein to aggregate into high molecular weight (HMW) structures and amyloid fibrils [77]. A linear correlation has been shown between insoluble α-synuclein aggregates and GCase activity loss in human sporadic PD midbrains. Moreover, certain disturbances in lipid homeostasis, that are the result of age-dependent decline in GCase activity, may result in altered protein lipid interactions and lead to lipid-stabilized α-synuclein and phosphorylated tau accumulation in neuronal vesicular membrane compartments [144].

### 4.2. Autophagic–Lysosomal Pathway, Protein Metabolism and GCase Interaction with Lysosome

It is well known that GCase deficiency results in disturbances in lysosomal function as well as in intracellular trafficking in neurons [18]. Some studies have shown that GCase deficiency resulting in GlcCer neuronal accumulation can promote α-synuclein oligomers formation. Mechanisms of this phenomenon are multifaceted. Supposedly, GCase decline causes an inhibition of lysosomal proteolytic functions and therefore affects α-synuclein degradation [145,146].

Another mechanism by which MT GBA1 alleles might impact α-synuclein accumulation is through blocking chaperon-mediated autophagy (CMA). When presented at the lysosomal surface, MT GCase can inhibit CMA and cause α-synuclein accumulation—CMA pathway substrate [20].

On the other hand, the pathogenic A53T and A30P-synuclein may also down-regulate their own degradation by binding to the lysosomal receptor for CMA pathway and hence become toxic [147].

Interestingly, primary substantia nigra dopaminergic neurons without the MT GCase CMA motif have been protected from neuronal death induced by MT GCase, which further supports the potential role of CMA in α-synuclein pathology [20].

Moreover, in GBA-PD brains, the fraction of GBA that fails to fold in the ER is targeted to the lysosomes, which has been shown to inhibit lysosome-associated membrane protein 2 (LAMP2A) multimerization, subsequently blocking degradation of other CMA substrates, including α-synuclein. The described processes lead to disturbance in proteostasis, α-synuclein aggregation and thereby contribute to further PD pathology progression [20,148].

It has been demonstrated that upregulation of GCase activity by a small molecule GCase modulator S-181 in induced pluripotent stem cells (iPSC)-derived dopaminergic neurons can ameliorate α-synuclein accumulation, lipid substrate accumulation, dopamine oxidation and lysosomal dysfunction in both GBA1-linked and non-GBA1-linked PD cases. Hence, regulating GCase activity may be a promising target to reduce the lysosomal dysfunction and toxic oxidized dopamine accumulation in the affected dopaminergic neurons [149].

### 4.3. Mitochondrial Dysfunction

It is known that, apart from lysosomal dysfunction, cells expressing GBA1 mutations also show altered mitochondrial homeostasis and dysregulation in calcium levels. Interestingly, studies on postmortem anterior cingulate cortical tissue from PD patients with heterozygous GBA mutations suggested that GBA mutation may impair mitochondrial function via mitophagy inhibition. This alteration prevents cells from discarding dysfunctional mitochondria and leads to their accumulation [150,151].

In primary neurons and astrocytes from a neuronopathic GD mouse model, dysfunctional mitochondria accumulate in cells due to a dysfunction of protein and organelle degradation machinery. It is suggested that these autophagy defects and subsequent buildup of dysfunctional organelles and protein aggregates could importantly impact the course of disease in cells with GBA mutations [152,153].

Studies conducted on cell cultures and GBA knockout GD mouse models suggest a loss-of-function mechanism by which GBA mutations impair mitochondrial activity associated with GD [154]. Nevertheless, the mechanism by which heterozygous GBA mutations impair mitochondrial function is still unclear.

### 4.4. Neuroinflammation

It has been shown that PD patients with GBA mutations have increased plasma levels of some inflammatory molecules, including some monocyte markers (such as IL-8, MCP1, stem cell factor, pulmonary and activation-regulated chemokine (PARC) and macrophage inflammatory protein 1α (MIP1α)) β [155], in comparison with sporadic PD cases. Hence, neuroinflammation could also be implicated in PD pathology in patients with GBA mutations [156]. Some studies also show that the above-mentioned faulty lysosomal storage may lead to activation of the NLRP3 inflammasome in Gaucher macrophages; this supports the neuroinflammatory mechanism by which GBA mutations may contribute to exacerbating PD pathology at the molecular level [157]. However, the mechanisms and function of microglial GCase need to be further investigated to fully understand how glial cells may affect the course of PD in patients with GBA mutations.

### 4.5. Glucocerebrosidase and LIMP-2

The role of the lysosomal integral membrane protein type-2 (LIMP-2), which mediates the transport of GCase to lysosome is also unclear and little is known about its molecular interactions with GCase. In PD fibroblasts, it has been discovered that the GBA1 mutation (particularly N370S) may impact proper LIMP-2 function via altered lysosomal cholesterol accumulation [158]. It has been shown that mice with knocked-out *SCARB2* gene exhibit features such as upregulated levels of GluCer, accumulation of α-synuclein and dopaminergic neurodegeneration [159]. Nevertheless, further research is needed to characterize the connection between LIMP-2 function and GBA mutations in humans [158].

## 5. Conclusions

GBA1 mutations constitute one of the highest risk factors for developing PD and DLB, which are the most prevalent α-synucleinopathies. It is known that either type of GBA1 mutation or its localization may critically impact the course of GD and the related pathologies. For instance, in comparison with patients with PD who do not carry GBA1 mutations, GBA1-PD patients exhibit more severe and frequent motor and non-motor PD symptoms, as well as reduced survival rate. Hence, GBA1 variants prevail as an intriguing subject to investigate in the context of α-synucleinopathies. However, some questions remain unanswered, such as if there is a correlation between GBA1 mutations and MSA. The most abundant *GBA1* gene mutations are N370S or N409S, L444P/L483P and E326K/E365K. They are known to impact GCase activity and affect the pathways in which it is involved. As described earlier in this article, mechanisms by which GBA1 mutations affect the severity of α-synucleinopathies severity occur through various molecular events, such as decreased GCase activity and disturbances in the MT GCase trafficking from the ER to the lysosome, which result in the accumulation of various products. Subsequently, perturbations in GCase transport affect ALP and CMA pathways. Moreover, GCase dysfunction may initiate ER stress conditions and trigger the activation of the UPR signaling pathway. The following events such as mitochondrial dysfunction, disturbances in lipid homeostasis and neuroinflammation are also proposed to further aggravate α-synuclein accumulation. In sum, considering the vital role that GCase may play in α-synucleinopathies pathogenesis, studying the pathways in which it is involved could result in a better understanding of these incurable diseases and offer a new approach to their treatment. Especially, personalized targeted therapies are of utmost interest in the case of various genetic variants of GBA1, either in the potential treatment of GD or in α-synucleinopathies. Furthermore, the underestimated role of GBA1 mutations in parkinsonian patients should be taken into consideration in applying novel therapeutic approaches. As the presence of MT GBA1 often results in earlier symptom onset and more severe disease course, it may be essential to distinguish such PD patients and evaluate them for the presence of GBA1 mutation; this could provide an opportunity for the application of modern, targeted therapy, that could be more effective in case of genetically affected patients.

## Data Availability

Data sharing not applicable.
